# Regression on imperfect class labels derived by unsupervised clustering

**DOI:** 10.1093/bib/bbaa014

**Published:** 2020-03-03

**Authors:** Rasmus Froberg Brøndum, Thomas Yssing Michaelsen, Martin Bøgsted

**Keywords:** Clustering, cancer, statistics, machine learning, survival analysis

## Abstract

Outcome regressed on class labels identified by unsupervised clustering is custom in many applications. However, it is common to ignore the misclassification of class labels caused by the learning algorithm, which potentially leads to serious bias of the estimated effect parameters. Due to their generality we suggest to address the problem by use of regression calibration or the misclassification simulation and extrapolation method. Performance is illustrated by simulated data from Gaussian mixture models, documenting a reduced bias and improved coverage of confidence intervals when adjusting for misclassification with either method. Finally, we apply our method to data from a previous study, which regressed overall survival on class labels derived from unsupervised clustering of gene expression data from bone marrow samples of multiple myeloma patients.

## Introduction

In biomarker studies it is popular to perform an unsupervised clustering of high-dimensional variables like genome-wide screens of Single Nucleotide Polymorphisms (SNPs), gene expressions and protein data and regress for example treatment response, patient recorded outcome measures, time to disease progression or overall survival on these potentially mislabeled clusters. It is well known from the statistical literature that errors in continuous and categorical covariates can lead to loss of important information about effects on outcome [1]. However, to our surprise this is often ignored when regressing outcome on classes identified by unsupervised learning, which might lead to important clinical effect measures being overlooked [2–6].

We suggest to cast the problem as a covariate misclassification problem. This leaves us with a concourse of possible modeling and analysis options, see for example the book by Carroll *et al.* [1] or the recent review by Brakenhoff *et al.* [7]. A general approach, with good statistical properties, is to maximize the likelihood of a latent variable model joining the regression and classification models [8, 9]. Drawbacks to this approach are the following: (i) this process does not mimic the workflow of the biologists, for whom the basic question is to identify important biological processes and next to relate the clusters to clinical consequences; (ii) upon parameter estimation cluster membership needs *post hoc* to be estimated by e.g. the maximum *a posteriori* probability, whereby direct connection to the regression parameter is lost; (iii) this approach requires a statistical model of the clustering process, leaving out the possibility to combine popular unsupervised clustering algorithms, such as hierarchical clustering, with popular parametric regression models like generalized linear models and Cox’s proportional hazards model.

Due to its generality, we instead chose to study the two-stage modeling process. A number of *ad hoc* methods have been developed in various specific settings, which could be adapted to this situation. Examples include matrix methods [10], regression calibration (RC) [11], pooled estimation [12], multiple imputation [13], corrected score estimation [14] and simulation and extrapolation (simex) [15–17]. Among these methods, both simex and RC have become useful tools for correcting effect estimates in the presence of additive measurement error. RC can be applied to both continuous and categorical covariates, while the simex idea has been extended to the misclassification simex (mcsimex) method for correcting effect estimates in the presence of errors in categorical covariates [18]. In this paper, we chose to focus on these methods and compare them because of their generality, simplicity and direct applicability.

The article is organized as follows. In Section 2, we detail the statistical setting and outline the RC and mcsimex methods. Simulation studies in Section 3 show the performance of our proposal. In Section 4 we apply the mcsimex and RC approaches to data from a study on unsupervised clustering of gene expression data from cancer patients by Zhan *et al.* [5]. The results of the paper are discussed in Section 5 followed by computational details in Section 6.

## Statistical setting

### The naïve method

Assume we have $n$ i.i.d. realizations $\{(y_i,{\boldsymbol z}_i,h_i), i=1,\ldots ,n\}$ of $(Y,{\boldsymbol Z},H)$ where $Y$ is the outcome and ${\boldsymbol Z} \in \mathbb{R}^p$ are features that cluster the observations into unobserved classes $H \in \{1, 2,..., m\}$. As we do not directly observe the classes $H$, we use an unsupervised clustering method to infer the classes based on the features ${\boldsymbol Z}$ and coin them $H^*$. The misclassification error is characterized by the $m\times m$ misclassification matrix $\Pi = [\pi _{ij}]_{m\times m}$, which is defined by its components

(1) \begin{align*} \pi_{ij} = P(H^*=i \;|\; H = j),\; i,j=1,\ldots,m. \end{align*}

Assume that we want estimates of the effect sizes ${\boldsymbol \beta }$ of each of the unobserved $m$ components, using e.g. a generalized linear model

(2) \begin{align*} g(E (Y \;|\; H=h)) = \boldsymbol{x}_h^\top\boldsymbol{\beta}, \end{align*}

where ${\boldsymbol \beta } = (\beta _1,\ldots ,\beta _m)^\top $, ${\boldsymbol x}_h = {\boldsymbol e}_1 + {\boldsymbol e}_h {\mathbb{1}}[h \not = 1]$ is encoded by treatment contrasts, $g$ is the link function and $Y$ is assumed to be generated from an exponential family distribution [19]. Optimization of the log-likelihood of the generalized linear model based on the assumed i.i.d. data $y_1 | h^*_1,\ldots ,y_n | h^*_n$, i.e. replacing the indicator functions in Equation (2) with indicators for the inferred classes, gives the estimate $\hat{{\boldsymbol \beta }}(\{(y_i,h^*_i), i=1,\ldots ,n\})$ [19]. This procedure will in the following be referred to as the naïve method.

It is important to notice that maximum likelihood estimation under the naïve method is estimation of a misspecified model, and that convergence of estimates under misspecified models is ensured [20]. It is, however, well known that estimating ${\boldsymbol \beta }$ by the naïve method leads to a biased estimate [18]. In general we will denote the limit of a maximum likelihood estimate by ${\boldsymbol \beta }(\Pi )$ for a model misspecified by the misclassification matrix $\Pi $.

### Regression calibration

RC is a simple approach that can be applied in many situations, see e.g. Carroll *et al.* [1] for a detailed introduction. The idea behind RC is that the misclassified variable $H^*$ can be replaced by the expected value of the true variable given the observed but misclassified variable, i.e. $E(H | H^*)$. For the application in the study at hand, this means that the indicator functions in (2) are replaced by the expected value of the indicators given the observed data, i.e. the posterior probability of the true class such that

$$\begin{equation*} \boldsymbol{x}_h = \boldsymbol{e}_1 + \sum_{h=2}^m\boldsymbol{e}_h P(H = h | \boldsymbol{Z} = z). \end{equation*}$$

In order to infer the expected value one must usually have a validation set with both values available, but we will instead assume that these probabilities are given as the posterior class probability from the clustering procedure, and can be inferred from e.g. Gaussian mixture models (GMM) or by using a fuzzy clustering procedure such as fuzzy $c$-means [21] instead of the hard clustering algorithm $k$-means.

### The mcsimex method

In order to formulate the *mcsimex* method, Küchenhoff *et al.* [18] defines the function $\mathcal{G}: [-1,\infty ) \rightarrow{\mathbb{R}}^m$ by

(3) \begin{align*} \mathcal{G}(\lambda) = \boldsymbol{\beta}(\Pi^{(1+\lambda)}), \end{align*}

where $\Pi ^{\lambda }$ can be expressed as $\Pi ^{\lambda } = E\Lambda ^{\lambda } E^{-1}$ via the spectral decomposition, with $\Lambda $ being the diagonal matrix of eigenvalues and $E$ the corresponding matrix of eigenvectors. For the function (3) to be well defined, we need to ensure the existence of $\Pi ^{\lambda }$ and that it is a misclassification matrix for $\lambda \geq 0$. Criteria for existence are given by Küchenhoff *et al.* [18].

We notice that $\mathcal{G}$ parameterizes the amount of misclassification, where $\mathcal{G}(-1)={\boldsymbol \beta }(I_{m\times m})$ corresponds to no misclassification, $\mathcal{G}(0)={\boldsymbol \beta }(\Pi ) $ corresponds to the present misclassification and $\mathcal{G}(\lambda )$ for $\lambda \geq 0$ corresponds to increasing misclassification. The fundamental idea behind mcsimex is to simulate $\mathcal{G}(\lambda )$ for increasing $\lambda \geq 0$ and then extrapolate back to $\lambda = -1$.

In a few situations explicit forms of $\mathcal{G}$ as a function of $\lambda $ can be calculated, but they tend to be unstable to estimate, wherefore mcsimex relies on finite-dimensional parametric approximations $\mathcal{G}(\lambda , \gamma )$, $\gamma \in{\mathbb{R}}^k$, of $\mathcal{G}(\lambda )$. One example from the R-package simex is the quadratic approximation $\mathcal{G}(\lambda , (\gamma _0,\gamma _1,\gamma _2))=\gamma _0+\gamma _1*\lambda +\gamma _2*\lambda ^2$ [22]. It is custom in the mcsimex literature to assume one either has the misclassification matrix at hand or it can be estimated from training data. Details for estimation of the misclassification matrix in the paper at hand are given in the sections on analysis of simulated and real data.

The mcsimex method (or algorithm) can now be formulated as:

**Algorithm [18]**

1. **Simulation of data with added noise.** For a fixed grid of values $1 < \lambda _1 <\cdots <\lambda _m$, simulate B data sets with increased noise, i.e. generate i.i.d. random variables (condition upon $\{(y_i,{\boldsymbol z}_i), i=1,\ldots ,n\})$
  $$\begin{align*} H^*_{b,i}(\lambda_k) \sim \textrm{Categorical}\left(\widehat{\Pi}^{\lambda_k}_{\bullet \hat{h}_i}\right), \end{align*}$$
  where $b=1,\ldots ,B$, $i=1,\ldots ,n$, $k=1,\ldots ,m$ and $\widehat{\Pi }^{\lambda _k}_{\bullet \hat{h}_i}$ is the $\hat{h}_i$’th column of $\widehat{\Pi }^{\lambda _k}$.
2. **Parameter estimation in simulated data.** For each level of increased noise obtain the mean parameter estimate by averaging over estimates from the $B$ data sets:
  $$\begin{align*} \hat{\boldsymbol{\beta}}({\lambda_k}) = B^{-1}\sum_{b=1}^B \hat{\boldsymbol{\beta}}(\{(y_i,h^*_{b,i}(\lambda_k)), i=1,\ldots,n\}). \end{align*}$$
3. **Fit a curve to mean parameter estimates.** Parameters for the curve,$\gamma $, are obtained by the least squares method:
  $$\begin{align*} \hat{\gamma} = arg\,min_{\gamma \in \Gamma} \sum_{i=0}^{k} \left(\hat{\boldsymbol{\beta}}({\lambda_k}) - \mathcal{G}(\lambda_k, \gamma)\right)^2, \end{align*}$$
  where $\lambda _0 = 1$ and $\hat{{\boldsymbol \beta }}({\lambda _0}) = \hat{{\boldsymbol \beta }}(\{(y_i,\hat{h}_i), i=1,\ldots ,n\})$ is the naïve estimator.
4. **Extrapolation.** The mcsimex estimate is then given by the extrapolation to $\lambda = -1$
  $$\begin{align*} \hat{\boldsymbol{\beta}}_{S} = \mathcal{G}(-1,\hat{\gamma}). \end{align*}$$

**Figure 1:**
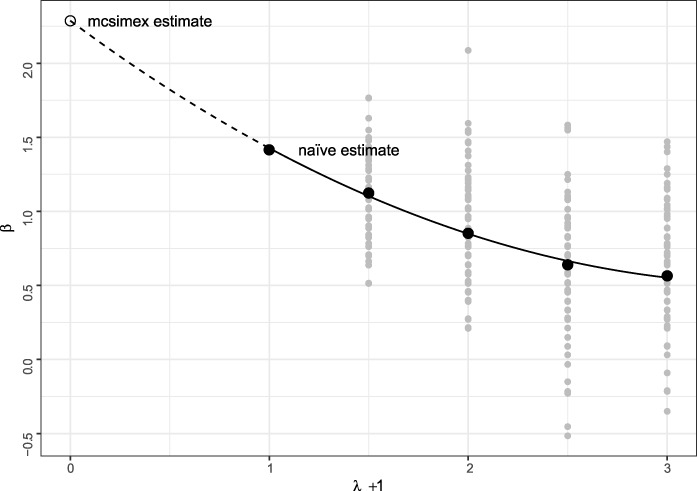
Illustration of the mcsimex method. Class labels were simulated from a binomial distribution with $P=0.5$ and noise was added to achieve a misclassification probability of $0.2$. A binary outcome with a true odds ratio of $\beta = 2$ was generated, and a logistic regression model was fit to the misclassified labels to get the naïve parameter estimate $\beta (\Pi )$. For each level of increased noise, $\lambda \in (0.5,1,1.5,2)$, $100$ simulated data sets were generated by misclassifying labels according to the misclassification matrix $\Pi ^{\lambda }$ and an estimate of $\beta $ was inferred by refitting the model (gray dots). A quadratic curve was fit through the naïve estimate and mean parameter estimates for each value of $\lambda $ (black dots). Finally, the mcsimex estimate was obtained by extrapolating the curve to the level of no noise, $\lambda = -1$.

The mcsimex method is illustrated in Figure 1

## Simulation studies

### Logistic regression

In this section, we investigate empirically how different sample sizes, imbalances between classes and clustering algorithms affect the effect estimates. For the simulations, we generate $n = 200$, $n=500$ or $n=1000$ independent samples from a two-class GMM, where the prior probabilities of class 1 are $\pi _1 = 5/10$ or $\pi _1 = 2/10$ to generate balanced or imbalanced classes, respectively, and set $\pi _2 = 1 - \pi _1$. Class 1 and 2 observations have bivariate normal distributions with means $\mu _1=(-1, 0)$ and $\mu _2=(1, 0)$, respectively, and a common identity covariance matrix. The outcome is modeled by a logistic regression with linear predictor ${\boldsymbol x}_h^\top{\boldsymbol \beta }$ where the intercept and class effect are given by $(\beta _1, \beta _2) = (-1,2)$.

Unsupervised clustering was performed using either GMM as implemented in the R-package mcclust [23], $k$-means from the base implementation in R or fuzzy $c$-means from the R-package e1071 [24]. We estimated ${\boldsymbol \beta }$ using either the true class labels, class labels inferred from unsupervised clustering with GMM or $k$-means, class probabilities for individual samples obtained as posterior class probabilities from GMM or fuzzy $c$-means and the naïve model corrected using the mcsimex method. The misclassification matrix for the mcsimex approach was inferred separately for each round of simulation. First, we inferred the class probabilities, means and variance–covariance matrices of the GMM or K-means clustering from the simulated data. Secondly, we simulated 100 000 bivariate normal samples from each of the inferred classes. Thirdly, for each of the 100 000 samples we predicted the class membership based on the simulated binary sample using respectively the maximum posterior probability of the classes or shortest distance to the class center given the estimated GMM or K-means clustering model from step one. Finally, we calculated the ratio of classified and misclassified predicted classes to the underlying simulated classes.

Each scenario was repeated 1000 times and the results are summarized by bias and coverage of the confidence intervals. Results from the balanced scenario $\pi _1 = 5/10$ are shown in Table 1. For ease of presentation, results of applying the RC approach to class probabilities from fuzzy $c$-means are presented under a $k$-means header. We see that using the true class labels there is a small bias in both of the estimated parameters and the coverage is close to $95\%$. When using the estimated class labels inferred from either the GMM or $k$-means clustering without taking misclassification into account, i.e. the naïve model, both parameters are biased and coverage is far from the assumed $95\%$. The bias is not alleviated by increasing the number of samples, the coverage is however smaller, due to a smaller standard error. When adjusting for misclassification using either RC or the mcsimex approach, both bias and coverage of the parameters are improved, and the improvement is similar across sample sizes and methods. Results from the imbalanced scenario are shown in Table 2. These also show smaller bias and better coverage for the adjusted models. Results are, however, better when using GMM than $k$-means. The $k$-means algorithm tends to estimate clusters of uniform size [25], leading to poor performance with imbalanced clusters, and since we used the fitted $k$-means clusters to infer the misclassification matrix this also becomes misspecified and the simex approach cannot fully correct for the added noise. Some work has been done to alleviate this bias in the $k$-means algorithm, e.g. using multicenters [26] or undersampling [27]. We did not pursue this further in the present paper, but just notice that one might instead use the GMM to infer clusters in the imbalanced case.

**Table 1:** Results from applying RC and simex to simulated data with balanced classes and a binary outcome. A logistic regression model was fitted with the true or inferred class labels, from either GMM or $k$-means with and without simex correction. Results from the RC approach with fuzzy $c$-means probabilities are presented under the $k$-means header. Simulations were done with $200, 500$ or $1000$ samples

		GMM			$k$ -means		
	True	Naïve	Simex	RC	Naïve	Simex	RC
**200**							
bias $\beta _1$	−0.01	0.34	0.07	0.04	0.34	0.07	−0.04
bias $\beta _2$	0.03	−0.69	−0.17	−0.08	−0.67	−0.13	0.08
coverage $\beta _1$	0.94	0.55	0.86	0.85	0.60	0.92	0.94
coverage $\beta _2$	0.94	0.39	0.90	0.90	0.39	0.93	0.94
**500**							
bias $\beta _1$	0.00	0.36	0.08	0.03	0.34	0.06	−0.03
bias $\beta _2$	0.00	−0.71	−0.15	−0.06	−0.69	−0.14	0.06
coverage $\beta _1$	0.96	0.28	0.88	0.90	0.28	0.92	0.94
coverage $\beta _2$	0.96	0.06	0.91	0.92	0.05	0.90	0.95
**1000**							
bias $\beta _1$	0.00	0.35	0.06	0.02	0.35	0.08	−0.03
bias $\beta _2$	0.01	−0.69	−0.12	−0.03	−0.69	−0.15	0.05
coverage $\beta _1$	0.95	0.08	0.88	0.89	0.05	0.88	0.94
coverage $\beta _2$	0.96	0.00	0.90	0.93	0.00	0.88	0.94

**Table 2:** Results from applying RC and simex to simulated data with imbalanced classes and a binary outcome. A logistic regression model was fitted with the true or inferred class labels, from either GMM, or $k$-means with and without simex correction. Results from the RC approach with fuzzy $c$-means probabilities are presented under the $k$-means header. Simulations were done with $200, 500$ or $1000$ samples

		GMM			$k$ -means		
	True	Naïve	Simex	RC	Naïve	Simex	RC
**200**							
bias $\beta _1$	−0.06	0.52	0.14	0.08	1.10	0.83	0.87
bias $\beta _2$	0.06	−0.70	−0.17	−0.07	−1.16	−0.74	−0.68
coverage $\beta _1$	0.96	0.55	0.81	0.8	0.02	0.24	0.16
coverage $\beta _2$	0.95	0.49	0.89	0.9	0.05	0.63	0.67
**500**							
bias $\beta _1$	0.00	0.57	0.14	0.14	1.09	0.82	0.86
bias $\beta _2$	0.01	−0.75	−0.16	−0.12	−1.15	−0.73	−0.65
coverage $\beta _1$	0.95	0.32	0.82	0.80	0.00	0.04	0.00
coverage $\beta _2$	0.95	0.20	0.89	0.90	0.00	0.31	0.42
**1000**							
bias $\beta _1$	−0.01	0.53	0.08	0.09	1.08	0.81	0.85
bias $\beta _2$	0.01	−0.71	−0.10	−0.08	−1.15	−0.72	−0.65
coverage $\beta _1$	0.94	0.16	0.86	0.82	0.00	0.00	0.00
coverage $\beta _2$	0.95	0.04	0.89	0.90	0.00	0.09	0.16

### Cox’s proportional hazards model

In survival analysis Cox’s proportional hazards model is often used as the model of choice for inferring the impact of covariates on the rate of events. The R package **simex**, used for this paper, did not previously support this model, which poses a problem for using mcsimex in survival analysis. This can be circumvented by using the Poisson approximation [28], but we chose instead to augment the source code of the **simex** package to include the coxph model class from the survival package [29, 30].

To test the performance of our implementation we performed a 2nd round of simulations. Simulations and class label inference were performed in the same manner as in Section 3.1, but instead of a binary outcome survival times were drawn from an exponential distribution with parameter $\lambda = \textrm{class}, \textrm{class} = 1,2$, giving a true hazard ratio of $2$, and censoring times were drawn from and exponential distribution with parameter $\lambda = 0.5$. Results from the simulations are shown in Tables 3 and 4. The results confirm the mcsimex and RC methods also reduce biases in the estimated parameters from Cox’s proportional hazards model. However, the improvement in bias as well as the coverage is better in the balanced scenario. Coverages are always smaller for the simex method than RC, which indicates that standard errors for the estimates are too small. This is likely caused by estimating them with the jackknife variance estimator in the simex package. This has previously been shown to underestimate the variance, so using an asymptotic variance estimate is preferred [31]. An asymptotic variance estimate for Cox’s proportional hazards model, incorporating misclassification, has to the best of our knowledge not yet been derived, but the problem can be alleviated by using a bootstrap approach to estimate the variance, which also adds the possibility to include additional variance resulting from estimation of the misclassification matrix [31]. However, the bootstrap approach drastically increases computational cost as the simex model has to be fitted for each bootstrap sample.

**Table 3:** Results from applying RC and simex to simulated data with balanced classes and survival outcomes. Cox’s regression model was fitted with the true or inferred class labels, from either GMM or $k$-means with and without adjustment for misclassification. Results from the RC approach with fuzzy $c$-means probabilities are presented under the $k$-means header. Simulations were done with $200, 500$ or $1000$ samples

		GMM			$k$ -means		
	True	Naïve	Simex	RC	Naïve	Simex	RC
**200**							
bias	0.05	−0.42	0.16	−0.01	−0.38	−0.01	0.12
coverage	0.96	0.68	0.91	0.92	0.72	0.92	0.94
**500**							
bias	0.02	−0.42	−0.07	−0.02	−0.41	−0.07	0.06
coverage	0.95	0.38	0.92	0.94	0.40	0.91	0.94
**1000**							
bias	0.01	−0.42	−0.07	−0.02	−0.42	−0.09	0.04
coverage	0.95	0.11	0.90	0.95	0.12	0.89	0.96

**Table 4:** Results from applying RC and simex to simulated data with unbalanced classes and survival outcomes. Cox’s regression model was fitted with the true or inferred class labels, from either GMM or $k$-means with and without adjustment for misclassification. Results from the RC approach with fuzzy $c$-means probabilities are presented under the $k$-means header. Simulations were done with $200, 500$ or $1000$ samples

		GMM			$k$ -means		
	True	Naïve	Simex	RC	Naïve	Simex	RC
**200**							
biasb	0.07	−0.40	0.03	0.05	−0.62	−0.36	−0.35
coverage	0.94	0.72	0.88	0.92	0.38	0.79	0.84
**500**							
bias	0.01	−0.43	−0.05	−0.04	−0.63	−0.39	−0.37
coverage	0.94	0.55	0.89	0.92	0.06	0.63	0.72
**1000**							
bias	0.01	−0.42	−0.05	−0.03	−0.63	−0.40	−0.39
coverage	0.95	0.33	0.92	0.93	0.00	0.42	0.51

## Cancer subclassification

Since the invention of high-dimensional gene expression profiling, just before this millennium, a popular task has been to perform unsupervised cluster analysis on such data to identify new subgroups and correlate these subgroups to biological information, clinical data and outcome. In this paper, we consider an example from subclassification of multiple myeloma (MM) by Zhan *et al.* [5]. MM is a malignancy of end stage B cells that expand in the bone marrow, resulting in anemia, bone destruction and renal failure. The data set contains 414 gene expressions sets from the MM patient’s bone marrow. The gene expressions were profiled on Affymetrix HGU133 Plus 2 arrays and exported to.CEL-files by the Affymetrix Genomics Console. To replicate the analysis we downloaded raw.CEL files from the GEO repository GS24080 and matched these by patient IDs to cases included in [5] and were able to match 407 out of the 414 cases. This was done since raw data were not available in the repository indicated in the original paper, so we resorted to a later study from the same group. Data were MAS5 normalized and filtered according to instructions in the original study [5] resulting in a data set with gene expressions for 2169 genes. This is a higher number of genes than the 1559 reported in the original paper, and is possibly caused by the different number of samples and/or slight differences in the MAS5 normalization procedure as implemented in the R package **affy** [32] and the Affymetrix Microarray Suite GCOS 1.1.

**Figure 2:**
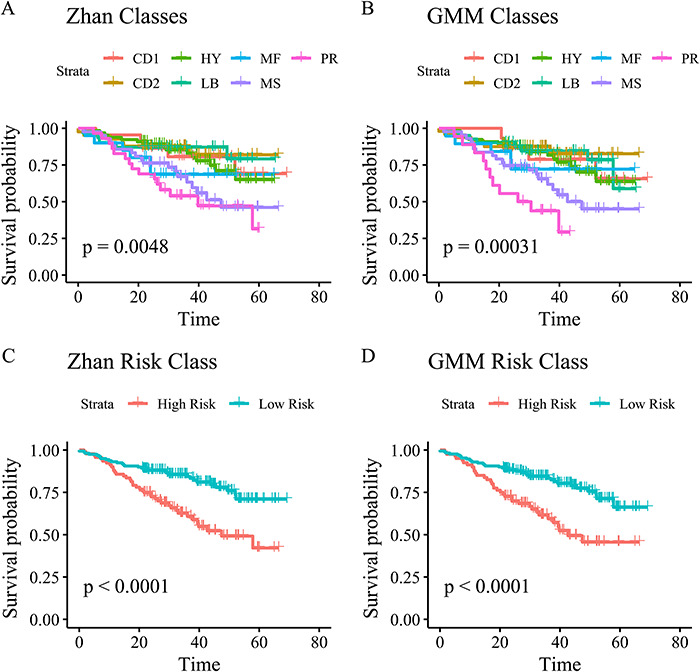
Kaplan–Meier curves for the identified classes, and risk groups in the original study by Zhan *et al.* (A, C) [5] and refitted with GMM classes (B, D). *P*-values arises from log-rank test of differences in survival.

After filtering, they performed hierarchical clustering on the remaining genes and chose to cut the tree, so seven clusters were formed. In order to mimic their analysis flow we identified seven subgroups by estimating a seven-component GMM. The classes were labeled according to the most similar class from [5] as shown in the confusion matrix in Table 5. The classes estimated from the GMM had an accuracy of 0.9 compared to the original classes. Kaplan–Meier curves for the original and GMM classes are shown in, respectively, panels A and B of Figure 2. These data confirm that a GMM reasonably well approximates the hierarchical clustering by Zhan *et al.* [5].

**Table 5:** Confusion matrix for class labels in the training data, accuracy = 0.9. Rows show original classes from Zhan *et al.* [5] and columns show clusters determined with the GMM

	1	2	3	4	5	6	7
CD1	$15$	$ 6$	$ 0$	$ 0$	$ 0$	$ 1$	$ 0$
CD2	$ 0$	$40$	$ 0$	$ 0$	$ 0$	$ 0$	$ 1$
HY	$ 0$	$ 2$	$63$	$ 0$	$ 0$	$ 0$	$ 0$
MS	$ 0$	$ 0$	$ 0$	$42$	$ 0$	$ 0$	$ 0$
MF	$ 0$	$ 0$	$ 0$	$ 0$	$19$	$ 1$	$ 0$
PR	$ 0$	$ 0$	$ 1$	$ 1$	$ 0$	$16$	$11$
LB	$ 0$	$ 0$	$ 2$	$ 0$	$ 0$	$ 0$	$29$

The seven classes were split into a low-risk group consisting of the CD1, CD2, HY and LB classes, and a high-risk group with the MF, MS and PR classes [5]. The accuracy of the original versus GMM risk groups was 0.98. Kaplan–Meier curves are shown in panel C of Figure 2 for the original classes and panel D for the GMM classes.

Cox’s proportional hazards model was employed to estimate the hazard ratio of the high- versus the low-risk group, but this analysis did not take any possible misclassification of the inferred classes from the unsupervised clustering into account. To investigate the impact of correction for misclassification we applied the RC and mcsimex method to the data at hand. For RC, class probabilities were obtained by summing posterior class probabilities for respectively high- and low-risk classes from a GMM fitted to the full data set. As shown in the simulation results the variance estimates of the built-in jackknife estimate for the mcsimex method are underestimated so we chose to do the analysis using bootstraps as well. We performed 1000 bootstraps iterations where at each step a sample of size $n=407$ was drawn with replacement from the available data. A GMM was fitted to the sample and the out-of-bag samples was used to infer the misclassification matrix by comparing the predicted class from the in-bag GMM model to the class obtained from the full data. Cox’s proportional hazards model was then fitted to the in-bag sample and the mcsimex model was applied using the estimated misclassification matrix. By estimating the misclassification matrix at each iteration of the bootstrap procedure we factor in the added variance from its estimation. Results from this, compared to RC and the naïve estimate along with results from using mcsimex with an average misclassification matrix from 1000 bootstraps, but only fitting the mcsimex model once, are shown in Table 6. For the average misclassification matrix we observed a misclassification probability of $0.07$ for the low risk group, and $0.13$ for the high-risk group. For the naïve models we see similar results for the original classes from [5] and the refitted classes from the GMM, as expected from the high accuracy for the risk groups. Using the RC method we find a similar estimate to the naïve approach. Investigation of the inferred class probabilities showed very little variation within class, i.e. values close to 1, explaining the similarity to the naïve estimate. This might be remedied by setting up a more elaborate bootstrapping scheme to infer individual class probabilities; this would however counteract the simplicity of the RC approach. For both simex approaches we find a higher point estimate for the hazard ratio of high versus low risk; confidence intervals are, however, wide and overlapping.

**Table 6:** Hazard ratio of high- versus low-risk groups in the training set from Zhan *et al.* [5] with the naïve and corrected models using the GMM classes

	HR	Lower 95	Upper 95	$P$ -value
Zhan classes - Naïve	$2.53$	$1.58$	$4.06$	$0.0001$
GMM - Naïve	$2.55$	$1.59$	$4.09$	$0.0001$
GMM - RC	$2.55$	$1.59$	$4.09$	$0.0001$
GMM - Simex (Average MC)	$3.19$	$1.71$	$5.95$	$0.0003$
GMM - Simex (Full bootstrap)	$3.12$	$1.72$	$5.66$	$0.0002$

## Discussion

In this paper, we documented a bias on effect estimates when regressing on misclassified labels arising from unsupervised learning. We also suggested a workflow for adjusting the effect estimates based on either the RC or mcsimex method. We had to extend existing software to appropriately handle regression based on time to event outcome. The effectiveness of the workflow was documented on simulated data, and we also shed new light on bias and variance of effect estimates in an existing cancer subclassification study.

The strength of the suggested workflow is essentially its general applicability and seemingly robustness. However, these advantages come at the cost of computational intensiveness of the Monte Carlo approach in mcsimex. For the RC approach computations requires little extra compared to the naïve approach as class indicators are simply replaced by class probabilities in the regression model. The analysis on true data did, however, suggest that in some instances this approach might be too simple.

As mentioned in Section 1 there exists a number of alternative methods to handle misclassified labels in regression models. Latent variable models seem most interesting as they are built on parametric models and maximum likelihood estimation. However, we have only come across one study dealing with the misclassification problem arising from unsupervised learning, but here a slightly different set-up is studied, as they formulate a latent variable model for class risk given measured class features and additional covariates [8].

In the light of our results, we encourage researchers to adjust for bias when regressing on potentially misclassified labels. We also encourage biomarker researchers to revisit previous studies, especially those that led to negative results when regressing upon misclassified labels.

## Computational details

All simulations and analyses were carried out by the statistical programming language R. For RC, class labels were simply replaced by the class probabilities, i.e regression was done on the probability of class 2, while the mcsimex function from the simex package was used as the main vehicle [22] for mcsimex analyses. This function contains functionality for mcsimex correction of regressions from the lm, glm, gam, nls, polr, lme and nlme functions. For the current study we extended the package to accommodate Cox’s proportional hazards model via the coxph function of the survival package [29, 30]. This extension was made by forking the source code of the simex package from https://github.com/cran/simex. The adapted code has been included in the simex package and is available at https://cran.r-project.org/package=simex

We utilized a number of other R and Bioconductor packages, notably the mclust and e1071 packages for clustering [23, 24]. For a complete list of packages see the R markdown document available at https://github.com/HaemAalborg/misClass, which details all steps in the analyses carried out in this paper.

**Key Points**

- Class labels from unsupervised clustering are prone to misclassification.
- Effect estimates based on these class labels may be biased.
- The bias can be reduced by correcting for misclassification with the generally applicable regression calibration and mcsimex methods.

## Notes

**Rasmus Froberg Brøndum** holds an MSc in Statistics, and a PhD in quantitative genetics. He currently works as a senior bioinformatician at Aalborg University Hospital, focusing on data-driven research in haematological malignancies.

**Thomas Yssing Michaelsen** is a PhD student at Aalborg University with in an interest in developing novel methods to analyze ‘omics’-data from microbial ecosystems. He holds an MSc in Biology, where the focus of his thesis was on implementing gene expression profiling as a routine clinical tool for prognosis and treatment guidance.

**Martin Bøgsted** is a Professor in Bioinformatics and Statistics at the Department of Clinical Medicine at Aalborg University and a Senior Statistician at the Department of Haematology, Aalborg University Hospital. He holds an MSc degree in Mathematics and Statistics as well as a PhD degree in Statistics. His main interest is in statistical issues related to translating high-throughput biological and big clinical data findings to clinical practice.

**The Haematology Research Laboratory** at Aalborg university hospital aims to explore the molecular mechanisms that underlie cellular function and contribute to human disease. In a multidisciplinary effort that spans molecular biology, medicine and biostatistics we aim at deconstructing cellular differentiation derived chemotherapy resistance as a driver of cancer progression in several hematological malignancies and translate these discoveries into new diagnostic, prognostic and predictive characteristics.
